# Supplementary material for: Gender differences in severity and parental estimation of adolescent’s pandemic-related stress in the United States
Source: PLOS Ment Health. 2024 Sep 3;1(4):e0000101. doi: 10.1371/journal.pmen.0000101 (PMC12798631; doi:10.1371/journal.pmen.0000101)
Supplement: S1 Table — This table presents each covariate used in the analysis with its definition and coding notes. (DOCX) [file pmen.0000101.s001.docx]

| **S1 Table. Covariates used in analyses** | |
| --- | --- |
| Age: | The PATH study data use two youth age categories: 12-14 and 15-17. We created a dummy variable where 1 represented a respondent aged 15-17 and 0 represented respondents aged 12-14. |
| Race/ethnicity: | The PATH study data categorized race into three categories: Black, White, and Other. Ethnicity was categorized as either Hispanic or non-Hispanic. We combined the race and ethnicity variables to create dummy variables for each category: Hispanic White, non-Hispanic White (reference), Hispanic Black, non-Hispanic Black, Hispanic Other, and non-Hispanic Other. For each dummy variable 1 represented a respondent being in the respective race/ethnicity category and 0 represented a respondent not being in the respective race/ethnicity category. |
| Body Mass Index (BMI): | BMI percentiles for each youth respondent were calculated by the PATH study from self-reported height and weight according to age and gender. |
| Physical activity: | Youth respondents are asked, “*During the past 7 days, on how many days were you physically active for a total of at least 60 minutes per day? (Add up all the time you spent in any kind of physical activity that increased your heart rate and made you breathe hard some of the time*)” Answer choices included, “*0 days,*” “*1 day,*” “*2 days,*” “*3 days,*” “*4 days,*” “*5 days,*” “*6 days,*” and “*7 days.*” We used this as a continuous variable ranging from 0-7. |
| Parental education: | Parent/guardian respondents are asked, “*What is the highest grade or year of school that you or your spouse/ guardian have completed?”* Answer choices included, “*Less than High School*” (reference), “*GED,*” “*High school graduate,*” “*Some college (no degree) or Associates degree,*” “*Bachelor’s degree,*” and “*Advanced degree.*” |
| Household income: | Parent/guardian respondents are asked, “*Which of the following categories best describes your total household income in the past 12 months?* *This is the total income before taxes of all persons in your household combined.*” Answer choices included, “*less than $10,000*” (reference), “*$10,000 to $24,999,*” “*$25,000 to $49,999,*” “*$50,000 to $99,999,*” and “*$100,000 or more.*” |
| Previous year anxiety: | Youth respondents are asked, “*When was the last time that you had significant problems with feeling very anxious, nervous, tense, scared, panicked, or like something bad was going to happen?*” Answer choices included, “*Past month,*” (reference) “*2 to 12 months ago,*” “*Over a year ago,*” and “*Never.*” This variable was taken from Wave 5. |
| Previous year depression: | Youth respondents are asked, “*When was the last time that you had significant problems with feeling very trapped, lonely, sad, blue, depressed, or hopeless about the future?*” Answer choices included, “*Past month,*” (reference) “*2 to 12 months ago,*” “*Over a year ago,*” and “*Never.*” This variable was taken from Wave 5. |
| Previous year overall mental health: | Youth respondents are asked, “*In general, how would you rate your...*  *mental health, which includes stress, depression, and problems with emotions?*” Answer choices included, “*Excellent*,” (reference) “*Very* *good*,” “*Good*”, “*Fair*”, and “*Poor.*” This variable was taken from Wave 5. |
| Sleep trouble: | Youth respondents are asked, “*When was the last time that you had significant problems with sleep trouble, such as bad dreams, sleeping restlessly, or falling asleep during the day?*” Answer choices included, “*Past month,*” (reference) “*Two to twelve months ago,*” “*Over a year ago,*” and “*Never.*” |
| TV stream time: | Youth respondents are asked, “*On an average weekday, about how much time do you spend watching television and streaming videos that*  *include commercials?*” Answer choices include, “*None*,” (reference) “*Less than 1 hour*,” “*1 to 2 hours*,” “*3 to 4 hours*,” “*5 to 6 hours*,” “*7 to 8 hours*,” “*9 to 10 hours*,” and “*11 hours or more*.” |
| Social distancing: | Youth respondents are asked, “*In the past 30 days, how often are you staying away from friends who live outside your home?*” Answer choices included, “*All the time*,” (reference) “*Most of the time*,” “*Sometimes*,” “*Rarely*,” “*Not at all*,” and “*I almost never saw friends in-person outside my home even before the*  *coronavirus*.” |
| Parental marital status: | Parent respondents are asked, “*What is your marital status? Are you now married, widowed, divorced, separated or never married?*” Answer choices include, “*Married*,” (reference) “*Widowed, divorced or separated*,” and “*Never married*.” |
| School performance: | Parent/guardian respondents were asked, “*How would you describe how (youth respondent) has performed at school in the past 12 months?*” Answer choices included, “*Mostly A’s,*” “*A’s and B’s,*” “*Mostly B’s,*” “*B’s and C’s,*” “*Mostly C’s,*” “*C’s and D’s,*” “*Mostly D’s,*” “*D’s and F’s,*” “*Mostly F’s,*” or “*Your child’s school is ungraded.*” We created a dummy variable where 1 represented respondents who reported “*mostly A’s*” through “*mostly B’s*” and 0 represented a respondent who reported “*Mostly B’s and C’s*” or lower as has been used previously [44,45]. Respondents who reported ungraded school performance were marked as missing and excluded from analyses. |
| Diagnosed with COVID-19: | Youth respondents are asked, “Has a doctor or other medical professional ever told you that you had the COVID-19 coronavirus?” Answer choices included, “*Yes*” or “*No*”. We created a dummy variable where 1 represented a respondent who reported “*Yes*” and 0 represented a respondent who reported “*No*”. |
| Past year tobacco use: | This variable was created by the PATH study and derived from a series of questions about past 12 months. The PATH study Wave 5.5 codebook lists the following description: “Wave 5.5 youth respondents who have smoked or used any of the following tobacco products within the past 12 months: cigarettes, e-products, traditional cigars, cigarillos, filtered cigars, hookah, pipe, smokeless tobacco, snus, dissolvable tobacco, bidis or kreteks.” The PATH study coded answers as either “*Yes*” or “*No*”. We created a dummy variable where 1 represented a respondent who had used tobacco in the past year and 0 represented a respondent who had not. |
| Past year substance use: | Youth respondents are asked a series of questions regarding substance use in the past year including: *In past 12 months,*   - *used prescription drug not prescribed to you: Ritalin or Adderall*, - *used prescription drug not prescribed to you: Painkillers* - *used prescription drug not prescribed to you: Sedatives or tranquilizers* - *used substance: Cocaine or crack* - *used substance: Stimulants like methamphetamine or speed* - *used substance: Heroin* - *used substance: Inhalants or solvents* - *used substance: Hallucinogens* - *used marijuana, hash, THC or grass* - *used alcohol at all*   Answer choices included, “*Yes*” or “*No*”. We created dummy variables for each substance inquired about where 1 represented a respondent who reported “*Yes*” and 0 represented a respondent who reported “*No*”. Because of a lack of representation for many of the substances inquired about, we removed substances with less than 10 instances per group, leaving: alcohol, painkillers, marijuana, and hallucinogens as covariates. |
