# Supplementary material for: Gender differences in severity and parental estimation of adolescent’s pandemic-related stress in the United States
Source: PLOS Ment Health. 2024 Sep 3;1(4):e0000101. doi: 10.1371/journal.pmen.0000101 (PMC12798631; doi:10.1371/journal.pmen.0000101)
Supplement: Attachment — Submitted filename: Response to reviewers letter - Pandemic Stress 2024 0507.docx [file pmen.0000101.s002.docx]

Response to reviewers letter: **Gender differences in severity and parental estimation of adolescent’s pandemic-related stress in the United States**

Reviewer #1 comments :

**Introduction:**
• **Summarize the introduction to provide a concise overview of the main points and objectives of the study.**

*We added a summary of the study and objectives to the first paragraph of the manuscript.*

**Discussion Section:**
**• Expand the discussion section to thoroughly explore the debate surrounding the topic and provide possible explanations for the findings or results presented in the study.**

*To date there is little research debating the topic of parental estimation of their children’s pandemic-related stress. We have added to the discussion possible explanations for our findings, along with a discussion of related research confirming the importance of parental support during the pandemic on adolescent resiliency and stress outcomes.*

**Covariant Table: should be included in supplementary data.**

*We have removed the covariate table from the main article and will submit separately as a supplementary table.*

Reviewer #2 comments: 

**Importance of the Study and Contribution to Knowledge:
The authors should provide a more profound elucidation of the significance of their study and its contribution to the existing body of knowledge within the field. An in-depth examination of the specific gaps in the literature that the study aims to address, and how it endeavors to fill them, would be invaluable. Furthermore, enhancing the contextualization of the study by elucidating how it builds upon previous research and introduces novel insights would be beneficial.**

*We added clarification of the dearth of literature assessing parental support dynamics in relation to their children’s pandemic-related stress, cited the only study we found on parental support in relation to their child’s pandemic-related stress, and clarified the specific gap in the literature that we address by looking at parental estimation of child pandemic-stress levels and differences by the child’s gender.*

**Methods:**
**Further exploration into the categories of analysis utilized, accompanied by a detailed explanation and clear differentiation between them, is recommended.**

*We added brief rationale for the three categories of covariates added to the models sociodemographics/psychographic-behavioral/substance use and information on their differentiation.*

**Providing insights into the rationale behind the selection of these categories and their alignment with the research objectives is paramount.**

*The paper includes a rationale for their selection and alignment with the objectives.*

**Additionally, inclusion of information regarding the psychometric properties and validation history of the utilized instruments, particularly focusing on sensitivity and specificity, is crucial.**

*We found no information on this related to the exact question used in the PATH survey, so we added this to the limitations section.*

**Moreover, a clearer exposition on the sampling methodology would be important. It is also essential to clarify how the secondary data were collected, including any ethical considerations involved in the original data collection process, as well as to outline the ethical considerations and protocols employed in the study.**

*We believe we explained this adequately in the second paragraph of the Methods section and we cite the relevant literature from the PATH study sampling methods and assessment of non-response bias. We further clarified that these surveys were conducted by telephone.*

**Results:
The duplication of results between the text and tables necessitates rectification, aligning with the expected standards of publications.**

*We deleted all in text specifications by model and replaced with a general summary of the findings across all models.*

**The analysis of results appears cursory and would benefit from a more comprehensive treatment.**

*We added to the discussion section a more comprehensive exploration of the findings and how they might apply to future research.*

**Furthermore, improving the presentation of results tables by revising the layout or format to enhance readability and facilitate interpretation is advised, as clear and well-organized tables significantly contribute to the overall clarity and effectiveness of communicating research outcomes.**

*We used the same format suggested to us in previous published manuscripts and believe the tables to be straight forward. If results for all covariates are preferred we can add those in but have been informed in the past that this is not appropriate and is more confusing to read through. Please provide more specific corrections if the table format for this journal is different than convention.*

**Discussion and Bibliography:
In the discussion section, it is essential not only to expound upon the findings but also to integrate them with existing scientific literature, particularly regarding other countries and studies.**

*We added more exploration of how our findings may integrate with existing research. We were unable to find studies from other countries assessing discrepancies in parental estimation of their children’s pandemic-related or overall stress not related to the pandemic. We add notes on the importance of future research in this area.*

**Updating the bibliography to encompass recent and pertinent sources, while refining the format of bibliographic citations, and considering the exclusion of gray literature, would enhance the scholarly rigor and relevance of the study.**

The bibliography has been updated to reflect the additions we made to the paper. Because this is a nascent research area, inclusion of all types of literature is paramount.
